# Biodistribution of ^10^B in Glioma Orthotopic Xenograft Mouse Model after Injection of L-para-Boronophenylalanine and Sodium Borocaptate

**DOI:** 10.3390/biomedicines9070722

**Published:** 2021-06-23

**Authors:** Natalya V. Gubanova, Alphiya R. Tsygankova, Evgenii L. Zavjalov, Alexander V. Romashchenko, Yuriy L. Orlov

**Affiliations:** 1 Institute of Cytology and Genetics, Siberian Branch Russian Academy of Sciences, 630090 Novosibirsk, Russia; zavjalov@bionet.nsc.ru (E.L.Z.); arom@bionet.nsc.ru (A.V.R.); orlov@bionet.nsc.ru (Y.L.O.); 2 Nikolaev Institute of Inorganic Chemistry, Siberian Branch Russian Academy of Sciences, 630090 Novosibirsk, Russia; alphiya@niic.nsc.ru; 3 Department of Natural Sciences, Novosibirsk State University, 630090 Novosibirsk, Russia; 4 Agrarian and Technological Institute, Peoples’ Friendship University of Russia, 117198 Moscow, Russia; 5 The Digital Health Institute, I.M. Sechenov First Moscow State Medical University of the Ministry of Health of the Russian Federation, 119911 Moscow, Russia

**Keywords:** glioma, boron neutron capture therapy, BNCT, L-para-boronophenylalanine, BPA, sodium borocaptate, BSH, mouse model

## Abstract

Boron neutron capture therapy (BNCT) is based on the ability of the boron-10 (^10^B) isotope to capture epithermal neutrons, as a result of which the isotope becomes unstable and decays into kinetically active elements that destroy cells where the nuclear reaction has occurred. The boron-carrying compounds—L-para-boronophenylalanine (BPA) and sodium mercaptoundecahydro-closo-dodecaborate (BSH)—have low toxicity and, today, are the only representatives of such compounds approved for clinical trials. For the effectiveness and safety of BNCT, a low boron content in normal tissues and substantially higher content in tumor tissue are required. This study evaluated the boron concentration in intracranial grafts of human glioma U87MG cells and normal tissues of the brain and other organs of mice at 1, 2.5 and 5 h after administration of the boron-carrying compounds. A detailed statistical analysis of the boron biodistribution dynamics was performed to find a ‘window of opportunity’ for BNCT. The data demonstrate variations in boron accumulation in different tissues depending on the compound used, as well as significant inter-animal variation. The protocol of administration of BPA and BSH compounds used did not allow achieving the parameters necessary for the successful course of BNCT in a glioma orthotopic xenograft mouse model.

## 1. Introduction

Therapy for malignant tumors in the human brain is rather challenging. Despite considerable efforts to develop new drugs and treatments, patients’ mean overall survival has increased by only three months (from 12 to 15 months). Their invasive nature and location in vital regions of the human brain make these types of tumors difficult for surgery; however, adjuvant therapy does not provide the desired results. Therefore, the elaboration of targeted radiotherapy methods is a promising approach for brain tumor therapy [1]. One such treatment option may be boron neutron capture therapy (BNCT).

BNCT exploits the ability of the isotope ^10^B to capture thermal neutrons and produce 478 keV *γ* rays, ^4^He particles and ^7^Li recoil ions, the latter two have high-linear energy transfer properties and a high biological effectiveness relative to photons. The range of these particles in tissue is restricted from 4 to 8 µm, thus limiting their effects to one cell diameter. Therefore, if ^10^B can be delivered selectively to tumor cells, the short range of these charged particles would offer the potential for targeting radiation to individual tumor cells with high biological efficacy. A boron delivery agent for BNCT would be required to fulfil all of the following conditions: (a) non-toxic at a clinically useful dose; (b) achieve at least 10–30 µg ^10^B per gram of tumor; (c) high tumor/brain and tumor/blood concentration ratios; (d) rapid clearance from blood circulation and normal tissues but persistence in tumor; (e) water solubility; and (f) chemical stability [2,3]. Sodium mercaptoundecahydro-closo-dodecaborate (BSH) and L-para-boronophenylalanine (BPA) are two compounds currently undergoing investigation in clinical trials; however, neither of them fully meets these requirements [4].

It has been shown in clinical trials that the boron uptake in gliomas of different patients may differ significantly. BSH biodistribution studies have suggested that the primary mode of the selective BSH distribution is passive diffusion from blood to tumor tissue via the disrupted blood–brain barrier (BBB). In contrast to BSH, BPA is actively transported through the tumor cell membrane due to the elevated expression of L-type amino acid transporters in tumor cells [5]. Despite numerous investigations, inconclusive information is available regarding these boron compounds’ metabolism and uptake in normal tissues and orthotopic glioma xenografts. 

This study investigated the dynamics of the boron concentration as delivered by BSH and BPA in normal mouse tissues and intracranial grafts of U87MG cells as a necessary step for further planning of experimental glioma treatment by BNCT.

## 2. Materials and Methods

### 2.1. Orthotopic Xenograft Mouse Model

**Figure 1:**
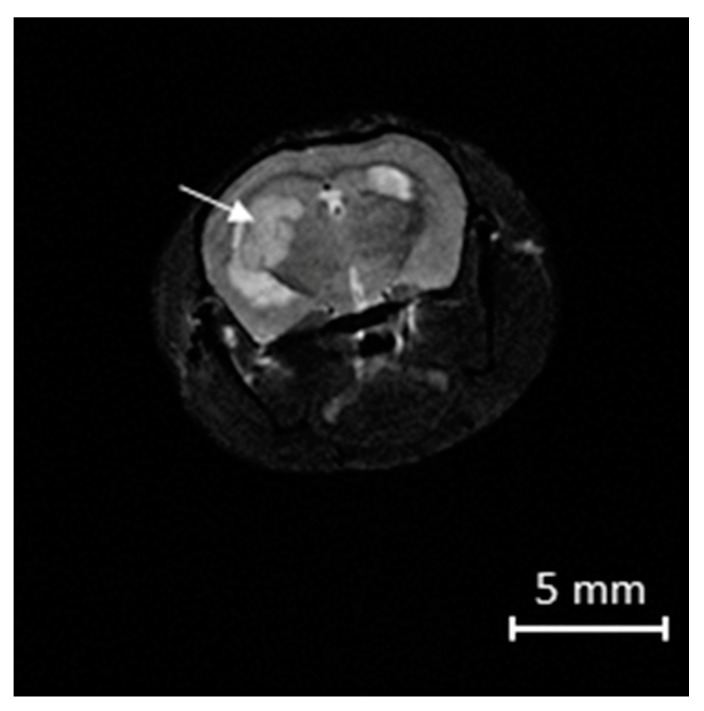
Axial MRI sections of the mice brains 4 weeks after xenotransplantation of U87MG cells. Arrow points to the tumor.

U87MG cells were grown on MEM Eagle growth medium (Sigma-Aldrich Co., St. Louis, MO, United States) supplemented with 10% fetal bovine serum (FBS) (HyClone, Cramlington, UK) using standard conditions (5% CO_2_, 37 °C). Male SCID mice aged 6–7 weeks were used. Mice were bred at the Centre for Genetic Resources of Laboratory Animals at the Institute of Cytology and Genetics, Siberian Branch, Russian Academy of Sciences, and housed under pathogen-free conditions. Their health status was investigated in accordance with the recommendations of the Federation of European Laboratory Animal Science Associations [6]. Animals had unlimited access to water and food (Ssniff Spezialdiäten GmbH, Soest, Germany). All experiments were performed in compliance with the protocols and recommendations for the correct use and care of laboratory animals (ECC Directive 86/609/EEC). Stereotactically guided intracranial injections in SCID mice were performed by injecting 500,000 U87MG cells at the junction between the cortex and striatum (coordinates, x = –2.0, y = 1.5, z = 2.4; bregma serving as the 0 point for x and y) [7]. Each group contained 8 animals. After 4 weeks, the tumors’ formation was confirmed by magnetic resonance imaging (MRI) on an ultra-high field tomograph, BioSpec 117/16 USR (Bruker Biospin GmbH, Ettlingen, Germany)—11.7 Tesla MRI (Figure 1).

### 2.2. Compounds

A distinctive feature of our work is the use of increased doses of boron delivery agents. Sodium mercaptoundecahydro-closo-dodecaborate (BSH, Na_2_^10^B_12_-H_11_SH) is a small hydrophilic anion [8]. L-para-boronophenylalanine (BPA, C_9_H_12_^10^BNO_4_) is a derivative of the neutral amino acid phenylalanine. Both compounds were purchased from Katchem Ltd. (Rez, Czech Republic). BPA was converted to a more soluble fructose complex by mixing BPA with fructose (Sigma-Aldrich Co., St. Louis, MO, United States) at a 1:1 molar ratio using a method reported by Coderre et al. [9]. The solution was stored at 4 °C overnight and then sterilized by filtration through a 0.2 µm filter. BSH was dissolved into PBS at a concentration of 18 mg/mL, sterilized by filtration through a 0.2 µm filter and kept in the injection syringe without gas available. The BPA compound was injected intraperitoneally at a dose of 1 g/kg body weight (b.w.)—equivalent to 48 mg^10^B/kg b.w. Due to its toxicity [10], BSH was administered subcutaneously into nuchal sites in a volume of 0.02 mL/g b.w.—equivalent to 360 mg/kg b.w. BSH and 205.6 mg ^10^B/kg b.w. No side effects due to drug administration were observed.

### 2.3. Boron Determination

Animals were sacrificed at 1, 2.5 and 5 h after the injection. Blood samples were collected on heparin before the animals were sacrificed, and samples of tumor tissue, normal brain (from the contralateral side of tumor), liver, kidney and skin from all the mice were obtained and weighed for the ^10^B determination. Boron concentration was analyzed by inductively coupled plasma atomic emission spectroscopy (ICP AES) on an iCAP-6500 high-resolution spectrometer (Thermo Scientific, Waltham, MA, USA). Registration of emission spectra was carried out under the iCAP conditions described in [11] after acid decomposition. Samples were injected with a peristaltic pump. The input system consisted of a sprayer and a cyclonic spray chamber (Tracey Spray Chamber) (PerkinElmer, Inc., Waltham, MA, USA). The concentration of ^10^B is expressed in µg per g (µg/g) of the tissue weight.

### 2.4. Statistical Analyses

Data analyses were carried out using Statistics 10 software (Stat Soft. Inc., Tulsa, OK, USA). We estimated the distribution parameters by a Shapiro–Wilk test. The mean, median and quartile values were determined. The relative ^10^B tissue concentration is expressed as the ratio of the absolute ^10^B concentrations in tissue and blood and was calculated for each animal. The ^10^B concentration ratios of the three time point groups were compared for each tissue separately using an exact Mann–Whitney test for independent groups for which we determined nominal two-sided *p*-values. Differences were considered statistically significant in the case of statistical tests with a *p*-value below the Bonferroni-adjusted nominal two-sided *p*-value.

## 3. Results

### 3.1. Dynamics of the Absolute Boron Concentration

Normality tests showed that datasets for the absolute value of the [^10^B] distribution in all tissues and groups did not correspond to a normal distribution in the given dataset. Therefore, for the statistical description of [^10^B], the values of the median and quartiles were chosen—the range of values within which 75% of the observed data fall. Table 1 presents the median [^10^B] values in various tissues at 1, 2.5 and 5 h after administration of BPA or BSH. 

**Figure 2:**
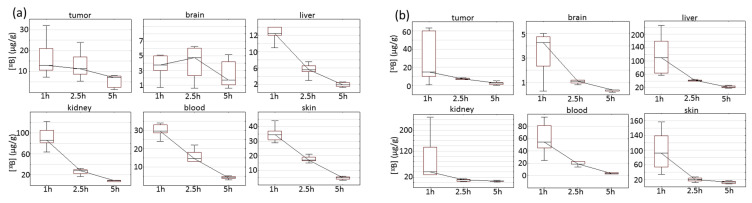
Box plots of absolute concentration of boron in different tissues at 1, 2.5 and 5 h after injection of BPA (**a**) and BSH (**b**). Box borders indicate 25th and 75th percentiles (interquartile range); lines within boxes indicate the median.

In general, there was considerable inter-animal variation in the ^10^B concentrations in all organs and tumors. The end-organ bioavailability was particularly high in kidneys for BPA and the liver for BSH. For the brain and tumor, the pharmacokinetics of BPA were different from other tissues (Figure 2). Boron concentrations did not change significantly in the brain during the entire observation time and in the tumor during the first 2.5 h, whereas in the blood, liver, kidney and skin, a statistically significant decrease in [^10^B], depending on time, was observed. The pharmacokinetics of BSH in the brain and tumor were more similar to those observed in normal tissues and significantly decreased with the observed time. Despite an almost 4-fold increase in [^10^B] at BSH administration compared with BPA (205.6 vs. 48 mg ^10^B per kg of body weight), a more than 7-fold increase in the boron concentration was observed in the liver but not in tumors. 

**Figure 3:**
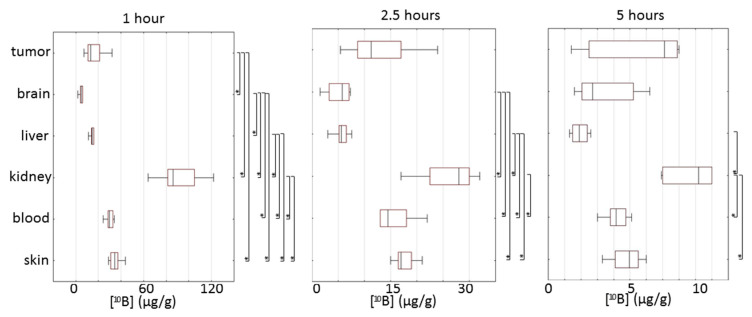
Box plots of absolute concentration of boron in different tissues at 1, 2.5 and 5 h after injection of BPA. Box borders indicate the 25th and the 75th percentiles (interquartile range); lines within boxes indicate the median. Significance of differences for 10B uptake in tissues was defined by a Mann–Whitney pairwise comparison followed by a Bonferroni post-test. * Statistical difference at *p* < 0.05.

A comparison of organ-specific ^10^B uptake showed that at 1 h after BPA administration, the highest difference in boron uptake by various tissues was observed (Figure 3). 

The highest boron concentration was detected in the kidneys, whereas the lowest values were recorded in the brain. The duration of BPA circulation in the blood not only reduced the concentration of boron in different tissues but also erased the differences between them. After 2.5 h, the concentration of boron in the tumor had no statistically significant differences compared to other tissues. The brain and liver had a similar concentration of boron, which was significantly lower than in the kidneys, blood and skin. After 5 h, significantly increased [^10^B] in the kidney compared to the liver, blood and skin was observed.

Thus, all tissues studied in this work, except for the brain and liver, during the first 2.5 h after BPA administration, can be classified as tissues at risk of damage as a result of BNCT. Measurements of [^10^B] after five hours of BPA administration showed safe [10B] in healthy animal tissues but low [^10^B] in tumors. Statistically significant Spearman’s correlations were found between the concentration of boron in the tumor and the brain (1 h after BPA injection) and the kidney and blood (2.5 h after BPA injection). After five hours, BPA injection tumor [^10^B] correlated with [^10^B] in the brain, liver, kidneys, skin, blood and mass of the tumor.

**Figure 4:**
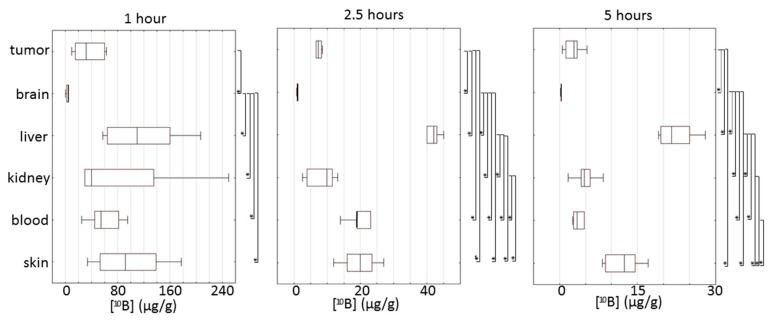
Box plots of absolute concentration of boron in different tissues at 1, 2.5 and 5 h after BSH injection. Box borders indicate 25th and 75th percentiles; lines within boxes indicate the median. Significance of differences for 10B uptake in tissue was defined by Mann–Whitney pairwise comparison followed by a Bonferroni post-test. * Statistical difference at *p* < 0.05.

The biodistribution of boron in tissues after BSH injection (Figure 4) reached the highest differentiation 2.5 h after injection, potentially due to subcutaneous administration of the drug. At 1 h, there was a significant scatter of indicators for all analyzed organs except for the brain, in which the boron content was the lowest for all of the time. [10B] in the tumor was significantly higher than the boron concentration in the brain but lower than or statistically indistinguishable from other tissues, particularly the more important ones, such as the blood and skin.

A significant correlation of [^10^B] was found between the skin and liver (1 h post-injection) and between the brain, blood and skin (2.5 h).

### 3.2. Dynamics of the Relative Boron Concentration

The relative boron concentration, which is expressed as the ratio of the tissue boron concentration to the blood boron concentration, plays a key role in the safety of BNCT. The tests for normality showed that the relative concentrations were closer to the normal distribution than the absolute ones, but they did not meet all criteria for normality. The data presented in Table 2 clearly demonstrate the insufficiency of the relative concentration of boron in the tumor for the safety of BNCT. A ratio above one (median 1.51) for the tumor was found only once—5 h after BPA injection. 

The ratio of 10B concentrations was significantly lower in the brain after injection with BSH than BPA over the duration of the entire investigation (Table 2), confirming the ability of BSH to penetrate into the tissue only through the destroyed blood–brain barrier. The relative concentration of boron in the brain significantly increased only by 5 h for both BPA and BSH. The leader in relative [10B] was the liver following BSH injections after 5 h (more than 7), whereas the absolute concentration was approximately 20 µg/g. For BPA, the highest values of this ratio were found in the kidneys 1 h after BPA injection (about 3), which decreased by 2.5 h and did not change significantly thereafter.

**Figure 5:**
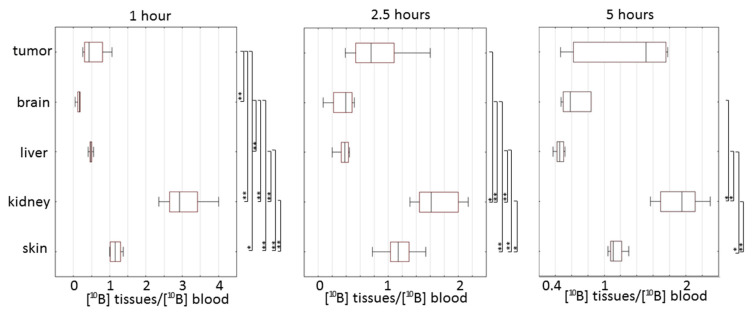
Box plots of [^10^B] ratios between tissues and blood in different tissues at 1, 2.5 and 5 h after injection of BPA. Box borders indicate 25th and 75th percentiles; line boxes indicate the median. Significance of differences for ^10^B uptake in tissue was defined by Mann–Whitney pairwise comparison followed by a Bonferroni post-test. * Statistical difference at *p* < 0.05. ** Statistical difference at *p* < 0.001.

A comparison of the relative concentrations of boron in different organs after BPA injection is shown in Figure 5. As with the absolute concentrations, the significance of differences was most pronounced in the first hour after BPA injection. It was during this period that the concentration ratio in the tumor was significantly higher than in the brain, was lower than in the kidneys and skin and did not differ from the liver. The brain showed the lowest value, and the kidneys displayed the highest one.

After 2.5 h, the relative concentration in the tumor differed only from the value of this indicator in the kidneys (below). After 5 h, when the relative concentration in the tumor was above 1, the differences for other organs finally leveled out. The only statistically significant Spearman correlation was found for the relative concentrations of boron in the brain and tumor 1 h after BPA injection.

**Figure 6:**
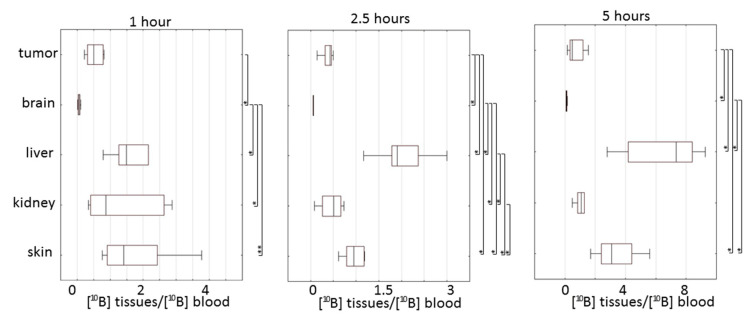
Box plots of [^10^B] ratios between tissues and blood in different tissues at 1, 2.5 and 5 h after injection of BSH. Box borders indicate 25th and 75th percentiles; lines within boxes indicate the median. Significance of differences for ^10^B uptake in tissue was defined by Mann–Whitney pairwise comparison and a subsequent Bonferroni post-test. * Statistical difference at *p* < 0.05. ** Statistical difference at *p* < 0.001.

After BSH treatment (Figure 6), there was a significant excess of the relative concentration of boron in the tumor compared to the brain over the time duration of the entire observation, and a significantly reduced tumor relative concentration in comparison with the liver and skin at 2.5 and 5 h. The only significant correlation was found for the relative concentrations of boron in the tumor and liver 2.5 h after BSH injection.

## 4. Discussion

The efficacy and safety of boron neutron capture therapy (BNCT) are proportional to the absolute and relative concentrations of boron in the tumor. A large number of works have been devoted to searching for a protocol for the administration of boron-carrying compounds to achieve the required absolute and relative boron concentrations. BPA and BSH were injected separately, sequentially and simultaneously; drug concentrations varied, and different administration routes were tested (intravenously, through the carotid artery, with damage to the BBB, etc.) [12,13].

A distinctive feature of our work is the use of an immunodeficient mouse model with orthotopic transplantation of human glioblastoma U87MG cells and increased doses of boron delivery agents. When planning the work, the results of clinical studies were considered, in which increasing the dose of BPA and time of intravenous infusion, followed by BNCT, improved the patient survival time [14,15,16]. Prolonged BPA circulation is thought to result in a more even micro-distribution of boron throughout the tumor [17]. In our study, BPA was injected intraperitoneally (1 g/kg body weight), or BSH was administered subcutaneously at the maximum permissible dose (360 mg/kg). We assumed that due to the high dose, the blood boron concentration would be high for a longer time, therefore contributing to the enrichment of the tumor with boron.

The used protocol for injections of boron-containing compounds did not allow achieving the parameters that corresponded to the conditions necessary for the successful course of BNCT.

A concentration higher than 20 µg/g in the tumor was found after 1 h of BSH injection (from 15 to 60 µg/g), but in the blood, this parameter was approximately 2-fold higher (from 30 to 135 µg/g), which would inevitably result in blood vessel damage after BNCT [18]. Therefore, BNCT at an absolute boron concentration in the tumor of 10 μg/g is acceptable, subject to safety treatment [17]. If we accept this assumption, then for the used protocol, the most suitable compound is BPA, and the time interval is 5 h after peritoneal injection. This time point is the only one in which the tumor/blood ratio is higher than 1 (~1.5), although the statistical significance of the differences in absolute and relative concentrations in the tumor, brain and blood has not been shown. Ideally, this value should be at least 3, but, in practice, a ^10^B concentration equal to the blood concentration is sufficient for safe treatment, as it has been shown for brain tumors [19,20]. The fact that the kidneys had a high relative boron concentration (~1.95) means that glioblastoma irradiation is still possible provided a suitable beam and a precise knowledge of whole-body dosimetry. The lack of statistical significance should be interpreted with caution because of the inter-animal variations in the pharmacokinetics of BPA and BSH, as we found for absolute and relative ^10^B concentrations for both compounds. Remarkable differences in the ^10^B concentration in the blood and normal tissues have already been shown in clinical trials and in a mouse model study using larger groups of animals (*n* = 15–32) [21,22,23,24,25], and our results confirm and supplement these studies with data on the boron concentration in glioma intracranial tumors in mice. The reasons for these differences are still elusive. Nevertheless, one aspect is clear—the observed variability implies a high level of uncertainty in determining the radiation dose from the boron neutron capture reaction in malignant and normal tissues. This point is particularly relevant for mouse models because the entire body of the animal is placed under the neutron flux, and high [^10^B] can cause significant normal tissue injury. Ideally, the ^10^B concentration in the tumor, normal tissue and blood should be known during irradiation. As there are no available methods for such measurements, in clinical trials, the concentration of ^10^B in the blood is measured, assuming a fixed ratio of [^10^B] between the blood and tissue. It has been shown that it is possible to safely administer the radiation dose using this procedure, but the observed variability between animals casts doubt on the method used to predict the concentration of ^10^B in tissues and, therefore, the absorbed dose for an individual. Thus, there is a high demand for methods for measuring the ^10^B concentration in vivo by positron emission tomography and magnetic resonance imaging.

In many studies, BSH and BPA are co-administered to increase the boron uptake by tumors because both compounds can access different subsets of tumor cells. It was previously shown that the injection of both compounds leads to an increase in the concentration of ^10^B in normal tissues and in the blood (additive effect) [25] that can lead to their injury. Possibly, two–three repeat injections of BPA according to the given protocol will allow the absolute and relative [^10^B] in the tumor to increase. In the work of Barth et al. [2], carried out on orthotopic glioma graft rat models, it was shown that the intracarotid administration of BPA and BSH makes it possible to increase, by almost 2-fold, the boron concentration in tumors (11 µg/g with intravenous injection compared with 20 µg/g intracarotid administration), and X-ray administration following BNCT increased rats’ mean survival times. Taking into account this study and numerous other studies [2,26,27], the feasibility of BNCT for adjuvant treatment has been suggested, combined with other methods.

The technological advances of the accelerator neutron sources’ development face a lack of effective compounds for BNCT, representing the main limiting factor [28]. In general, the presented results are consistent with the works of other researchers and supplement them, confirming the need to develop new drugs and dosimetry methods for neutron capture therapy.

## Figures and Tables

**Table 1:** ^10^B concentration after injection of BSH or BPA for different tissues. Data are presented as median and the 25th and 75th percentiles. Significance of differences was defined by Mann–Whitney pairwise comparison followed by a Bonferroni post-test. *^a^* Statistical difference according to 1-h group with the same compound (*p* < 0.05). *^a^*^’^ Statistical difference according to 1-h group with the same compound (*p* < 0.001). *^b^* Statistical difference according to 2.5-h group with the same compound (*p* < 0.05). *^b’^* Statistical difference according to 2.5-h group with the same compound (*p* < 0.001). * Statistical difference according to BPA group at the same time points (*p* < 0.05).

Time Post-Injection	^10^B Concentration in Tissues after Injection of BPA (µg [^10^B]/g)			^10^B Concentration in Tissue after Injection of BSH (µg [^10^B]/g)		
	1 h	2.5 h	5 h	1 h	2.5 h	5 h
tumor	13.0 [10.7; 21.0]	11.3 [8.7; 17.0]	7.1 [2.5; 7.9] *^ab^*	32.0 [15.0; 60.0]	7.4 [6.6; 8.3] * *^a^*	2.8 [1.2; 3.4] *^ab^*
brain	4.75 [4.0; 6.0]	5.8 [3.3; 7.0]	2.7 [2.1; 5.2]	4.25 [2.4; 4.8]	1.1 [0.95; 1.2] * *^a^*	0.4 [0.3; 0.4] * *^ab’^*
liver	14.5 [14.0; 16.0]	5.6 [5.2; 6.6] *^a’^*	1.9 [1.5; 2.4] *^a’b^*	110 [64.0; 160] *	42 [40.0; 43.0] * *^a’^*	21.5 [19.5; 25.0] * *^a’b’^*
kidney	86.0 [81.5; 105]	28.0 [22.5; 30.0] *^a’^*	9.2 [7.0; 10.0] *^a’b’^*	40.0 [30.0; 135]	9.9 [4.0; 11.5] * *^a’^*	4.8 [4.2; 5.9] * *^a^*
blood	29.5 [28.5; 33.0]	14.5 [13.0; 18.0] *^a’^*	4.2 [3.8; 4.8] *^a’b’^*	54.5 [45.0; 81.5] *	19.0 [19.0; 23] *^a’^*	3.4 [2.7; 4.8] *^a’b’^*
skin	34.5 [31.0; 37.0]	17.0 [16.5; 19.0] *^a’^*	5.0 [4.1; 5.5] *^a’b’^*	91.5 [53.5; 139] *	20.0 [16.0; 23.5] *^a’^*	12.5 [8.9; 14.5]* *^a’b^*

**Table 2:** ^10^B concentration ratios between tissues and blood after injection of BSH or BPA. Data are presented as median and the 25th and 75th percentiles and mean (±SD). Significance of differences was defined by Mann–Whitney pairwise comparison followed by a Bonferroni post-test. *^a^* Statistical difference according to 1-h group with the same compound (*p* < 0.05). *^a’^* Statistical difference according to 1-h group with the same compound (*p*< 0.001). *^b^* Statistical difference according to 2.5-h group with the same compound (*p* < 0.05). *^b’^* Statistical difference according to 2.5-h group with the same compound (*p* < 0.001). * Statistical difference according to BPA group at the same time points (*p* < 0.05).

Time Post-Injection	^10^B Concentration Ratios between Tissues and Blood after Injection of BPA			^10^B Concentration Ratios between Tissues and Blood after Injection of BSH		
	1 h	2.5 h	5 h	1 h	2.5 h	5 h
tumor	0.43 [0.31; 0.81] (0.6 ± 0.3)	0.75 [0.54; 1.1] (0.8 ± 0.4)	1.5 [0.63; 1.8] (1.3 ± 0.5) *^a^*	0.5 [0.32; 0.79] (0.52 ± 0.26)	0.42 [0.31; 0.45] (0.38 ± 0.13) *	0.48 [0.34; 1.2] (0.7 ± 0.5)
brain	0.17 [0.13; 0.20] (0.16 ± 0.06)	0.40 [0.22; 0.48] (0.35 ± 0.17)	0.58 [0.50; 0.84] (0.7 ± 0.3) *^a’b^*	0.05 [0.03; 0.09] (0.06 ± 0.03) *	0.05 [0.05; 0.06] (0.060 ± 0.004) *	0.09 [0.08; 0.14] (0.10 ± 0.03) * *^b’^*
liver	0.49 [0.46; 0.51] (0.5 ± 0.04)	0.38 [0.33; 0.43] (0.37 ± 0.08) *^a’^*	0.46 [0.43; 0.50] (0.53 ± 0.23)*^b^*	1.5 [1.3; 2.2] (1.9 ± 1.2) *	1.9 [1.8; 2.4] (2.0 ± 0.6) *	7.4 [4.2; 8.4] (6.5 ± 2.4) * *^ab’^*
kidney	2.9 [2.7; 3.4] (3.0 ± 0.6)	1.6 [1.4; 2.0] (1.7 ± 0.3) *^a’^*	1.95 [1.69; 2.10] (1.92 ± 0.26) *^a’^*	0.87 [0.41; 2.6] (1.2 ± 1.1) *	0.51 [0.26; 0.67] (0.47 ± 0.23) *	1.1 [0.84; 1.3] (1.2 ± 0.6) *
skin	1.15 [1.01; 1.30] (1.16 ± 0.15)	1.14 [1.03; 1.30] (1.16 ± 0.23)	1.11 [1.08; 1.22] (1.14 ± 0.09)	1.4 [0.92; 2.4] (1.8 ± 1.0)	0.95 [0.79; 1.2] (0.96 ± 0.22)	3.1 [2.4; 4.4] (3.4 ± 1.3) * *^b’^*
